# Psychiatric Disorders and Obesity in Childhood and Adolescence—A Systematic Review of Cross-Sectional Studies

**DOI:** 10.3390/children10020285

**Published:** 2023-02-01

**Authors:** Ioulia Kokka, Iraklis Mourikis, Flora Bacopoulou

**Affiliations:** 1 Outpatient Specialty Clinic for Obsessive Compulsive Disorder and Behavioral Therapy, First Department of Psychiatry, Medical School, National and Kapodistrian University of Athens, Eginition Hospital, 74 Vas. Sofias Ave, 11528 Athens, Greece; 2 Outpatient Specialty Clinic for Sexual Health, First Department of Psychiatry, Medical School, National and Kapodistrian University of Athens, Eginition Hospital, 74 Vas. Sofias Ave, 11528 Athens, Greece; 3 Center for Adolescent Medicine and UNESCO Chair in Adolescent Health Care, First Department of Pediatrics, Medical School, National and Kapodistrian University of Athens, Thivon & Papadiamantopoulou St., 11527 Athens, Greece

**Keywords:** psychiatric disorders, obesity, adolescents, children, mental health, anxiety, depression, mood disorders, psychosis

## Abstract

Obesity and psychiatric disorders have high prevalence and are both considered major health problems. Within the last decades, the rates of obesity have risen over 6%, while the prevalence of psychiatric disorders is over 12% for children and adolescents. The aim of this study was to systematically review the evidence regarding the relation of obesity and psychiatric disorders in childhood and adolescence. This review, based on the PRISMA guidelines, included cross-sectional studies published within the last decade, pertaining to the relation between psychiatric disorders and obesity in children and adolescents up to the age of 19 years. Studies on eating disorders were excluded. A total of 14 studies of 23,442 children and adolescents that investigated the relation of obesity with anxiety, mood disorders, and psychosis were included in this systematic review. Nine of the included studies reported a significant relationship between the psychiatric disorder under investigation and obesity. Understanding the nexus between obesity and psychiatric disorders in children and adolescents is of great importance, given the alarming increase in both conditions in youth. Such findings could facilitate the development and implementation of targeted interventions.

## 1. Introduction

Obesity and psychiatric disorders constitute major health problems, as they both demonstrate high prevalence and morbidity rates. Global obesity rates among almost 130 million children, adolescents, and adults have tripled since 1975, while in 2020 the World Health Organization reported that more than 340 million minors (5–19 years of age) were classified as obese [1]. More specifically, the rates of obese children and adolescents have climbed from 0.7% to 5.6% in males and from 0.9% to 7.8% in females [2]. There is still an unmet need for childhood and adolescent weight management, as studies have shown that some interventions are not as effective as expected. A recent review of 66 meta-analyses showed that unilateral interventions aiming solely on dietary patterns or physical activity—even though cost effective [3]—did not yield the expected results [4]. Childhood and adolescent obesity constitute a risk factor for several health issues [5] such as precocious puberty [6], menstrual irregularities and polycystic ovary syndrome in female adolescents [7], obstructive sleep apnea [8], and metabolic syndrome [9]. An overwhelming proportion (80%) of the obese adolescents will carry this issue into their adulthood [7]. Moreover, obesity can impact the mental health and quality of life of children and adolescents in a negative fashion as well. A large body of evidence suggests that obesity is linked to lower social and physical indicators of quality of life [8,9], as well as deteriorated dimensions of parent- and school-related psychological well-being [10,11]. A study has suggested that children and adolescents with extreme obesity report complaints that could simulate symptoms that are related to body dysmorphic disorders [12], while others suggest that they suffer from sleep disorders [13], as well as headaches. It appears that obesity may also affect the behavioral -internalized as well as externalized- patterns of children and adolescents [14]. There is evidence that obesity negatively impacts adolescents’ peer relationships [15], and may be associated with hyperactive behavior or overemotional symptomatology [16]. Studies have also studies obesity’s connection with more disturbing situations such as conduct difficulties, however no association was found [15]. Obesity has also been linked with experiences of discrimination. Research has shown that obese individuals are more likely to not complete their education, due to adverse experiences within the school setting [17], the so called weight bias, which refers to marginalization by peers or teachers [18]. More particularly, these experiences seem to be more frequent for girls compared to boys [19]. 

Like obesity, psychiatric disorders are considered a major contributor in the global burden of disease. A recent study that assessed the prevalence of mental illnesses among 204 countries and various age groups found that, between 1990 and 2019, the disability-adjusted life years (DALYs) due to mental disorders increased from 80.8 million to 125.3 million, constituting relevant diagnoses among the top ten leading causes of disease [20]. Regarding childhood, findings of a recent meta-analysis revealed that the prevalence of mental disorders during this life period in 11 countries, from 2003 and 2020, was 12.7%, with the most prominent being anxiety (5.2%), attention-deficit/hyperactivity (3.7%), oppositional defiant (3.3%), substance use (2.3%), conduct (1.3%), and depressive (1.3%) disorders [21]. Adolescence constitutes a transition period of life with drastic physical and psychosocial changes [22,23]. The contemporary developmental mismatch of adolescents’ biological and psychosocial maturation can render adolescence vulnerable to mental illnesses, such as anxiety and mood disorders, as well as eating disorders. According to the World Health Organization, mental health issues account for more than 15% of the adolescent burden of disease and injury, with depression appearing as the fourth leading cause of illness, and 20% of the adolescent population demonstrating some level of depressive symptomatology or anxiety [24]. 

Mental disorders such as anxiety [25], eating disorders [26], and depression [27] often co-occur with risk factors for poor physical health, such as obesity. Previous research suggests that individuals with persistent chronic mental illnesses are more susceptible to weight gain. Epidemiological and clinical studies have shown prevalent rates of co-occurrence of 20% to 60% for bipolar disorder [28,29], 30% to 70% for psychotic disorders [30,31], and 20% to 50% for depression [32]. An unquestionable iatrogenic link between mental illness and weight status is that of pharmaceutical intervention [33]. Despite the differences in weight gain among individuals, the most common psychopharmaceutical medications (antipsychotics, mood stabilizers, and antidepressants) may trigger some degree of weight increase through different metabolic functions [34]. With respect to underaged individuals relevant research has shown that psychopathology is more frequent in obese when compared to non-obese adolescents. For example, in a Dutch study with more than 20000 adolescents which implemented internet based measurements, obese individuals were more likely to report suicidal thoughts and attempts, and obese adolescents were more possible to receive a psychiatric diagnosis [35]. Moreover, a longitudinal study with 9374 adolescents, found that those who reported elevated depressive symptomatology were at higher risk of developing obesity and maintaining an unhealthy weight status [36]. What is a interesting finding is the results of a systematic review which has found a simultaneous increase of depressive symptomatology and obesity for minors aged 6 to 19 years, with a risk to maintain an unhealthy BMI up to 15 years later [37]. 

Within the past few decades, the scientific interest about the relation between obesity and psychiatric illness in youth has increased, with a number of systematic reviews conducted to synthesize the existing evidence [38,39,40]. However, these reviews have focused on specific disorders [41], or specific psychological traits of burden such as low self-esteem [42], rather than on a range of mental illnesses. Furthermore, given the heterogeneity of the existing studies on this research field, a gap regarding the relation between obesity and a range of psychiatric disorders in childhood and adolescence still exists. Therefore, the aim of this study was to provide in a systematic way the evidence regarding the relation of obesity and psychiatric disorders in children and adolescents and explore factors contributing to this association. 

## 2. Materials and Methods

The current review design was based on the PRISMA guidelines [43], to identify the most recent papers relevant to the research topic (details in supplementary material), without any primary data or use of any type of statistical method [44]. Stages of this research included:

▪ the research question formulation
▪ the extensive literature review of the topic
▪ the data extraction and evaluation, and lastly
▪ the data presentation and analysis.

**Figure 1:**
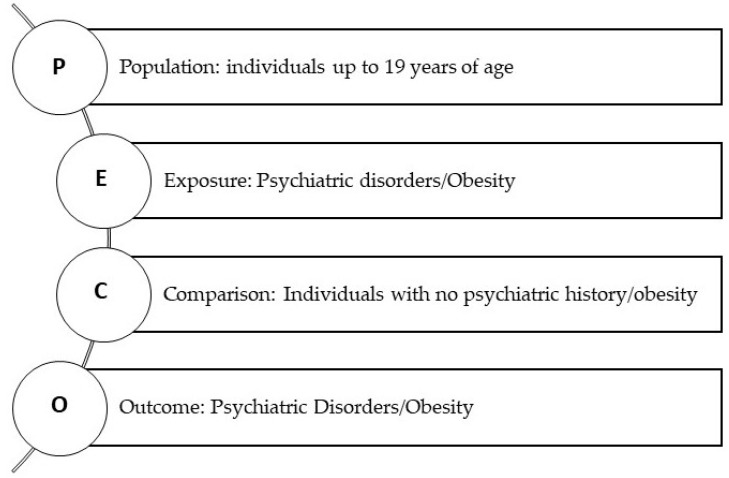
Diagrammatic presentation of the PECO framework.

The studies that were included in this review had to comply with specific eligibility criteria as indicated below. They could have investigated the impact of obesity on psychiatric well-being or vice versa. The PECO framework is presented in Figure 1.

### 2.1. Eligibility Criteria

Eligible studies for inclusion were cross-sectional studies published within the last decade in peer reviewed journals in the English language that investigated the relation between psychiatric disorders and body mass index. The studies had to include participants up to 19 years of age, according to the World Health Organization definition of the time period of childhood and adolescence [45]. Studies could include individuals irrespective of setting (clinical setting or general population). Eligible mental disorders were those based on standardized criteria, clinical diagnosis, self-reported diagnosis, or a score above a threshold on a validated scale/questionnaire. Obesity had to be defined according to body mass index (BMI) for the age and gender characteristics of each study’s sample [46,47]. Given the link between eating disorders and BMI, these disorders were excluded from the present review [26,48]. Studies including both underaged and adult samples, without providing separate data based on age, were not included. Other reviews or research protocols that did not provide adequate data were also excluded. 

### 2.2. Search Strategy

The Pubmed and PsycInfo databases were thoroughly searched. The literature review was conducted between the 21st of September and 15th of October 2022 by one investigator. The search terms used in both databases included the following:

“Psychiatric disorders” OR “mental illness” OR “anxiety disorders” OR “depressive disorders” OR “mood disorders” OR “psychosis”

AND

“Obesity” OR “overweight” OR “BMI” OR “body mass index” OR “excess weight”

AND

“Adolescent” OR “adolescence” OR “puberty” OR “childhood”.

The titles, abstracts, and keywords of each study were screened for eligibility. All included studies were evaluated according to the prespecified inclusion/exclusion criteria.

### 2.3. Data Extraction 

From each pertinent study specific data were extracted. These included the name of the first author, the year of publication, the sample size, basic demographic characteristics of the sample when provided (mean age, gender distribution), the primary diagnosis under investigation, and how this was established in each study. Lastly, the main findings of each study with respect to the research question were extracted. 

### 2.4. Data Evaluation

To assess each study’s quality level, the appraisal tool for cross-sectional studies (AXIS) was applied [49]. AXIS includes 20 items with each examining a different domain of the quality level of a study. These items refer to specific studies’ characteristics such as appropriateness of the design, the methodology, the statistical analysis, and internal consistency, The tool aims to assist the evaluation of observational and, more specifically, cross-sectional studies. Each item of the AXIS can be answered with “yes”, “no” or “I do not know”, yet this does not aim to conclude to a total score, due to acknowledged issues that emerge with such scores [50].

## 3. Results

### 3.1. Study Selection 

**Figure 2:**
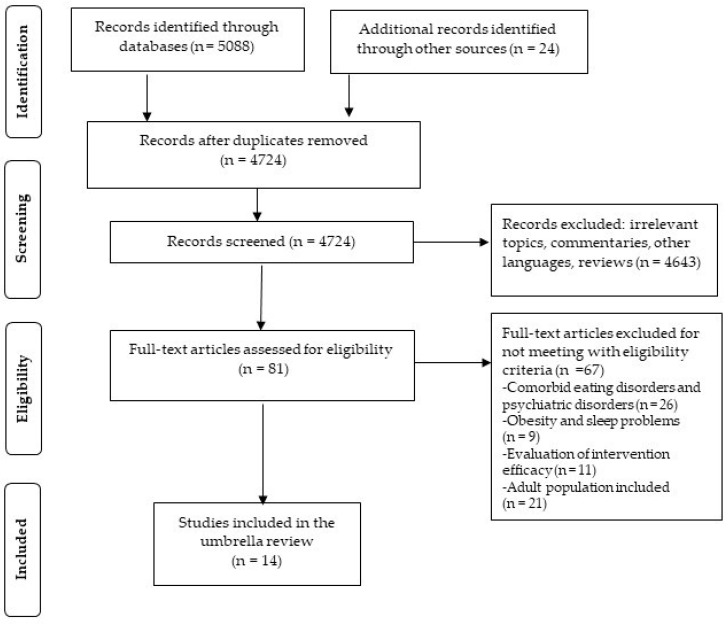
Study selection flowchart.

The initial search yielded a total of 5112 studies. After duplicates’ removal the remaining 4724 studies were screened by title. Following exclusion of irrelevant and unsuitable papers, 81 studies were thoroughly read. 26 of the studies were excluded for included comorbid eating and other psychiatric disorders, nine of them were excluded for reporting on the relationship of obesity and sleep disorders, 11 of them were not included as they evaluated the efficacy of interventions for weight management, while 21 of them included solely adults or mixed samples with minors and adults. After applying the inclusion/exclusion criteria, the final step of the literature search concluded in 14 cross-sectional studies. The complete screening procedure is presented in Figure 2. 

### 3.2. Basic Characteristics of Included Studies 

Studies were published between 2012 and 2021 and included 23,442 children and adolescents up to 19 years of age. The largest sample size included 12,507 participants, whereas the smallest 113 participants. Eight of the studies included solely adolescent samples, while the remaining studies included samples which consisted of both children and adolescents. Regarding the twelve studies, 55.8% of the participants were females, while two of the studies did not clarify participants’ gender distribution. Almost half of the studies (six out of fourteen) evaluated the psychiatric disorder under investigation with self-report instruments that provided clear cut-off diagnostic points, while the remaining eight performed clinical interviews based on specific diagnostic criteria (e.g., ICD-10, DSM-IV, DSM-V). Seven of the studies examined the relationship of obesity with depression [51,52,53,54,55,56,57], one with schizophrenia [58], and one with bipolar disorder [59]. Four explored the link between obesity and anxiety [54,57,60,61], while specifically social anxiety was assessed in two studies [49,50,62,63]. Lastly, one investigated the relationship of obesity and conduct disorder [49], while one investigated the existence of a psychiatric disorder based on the DSM-V criteria, without clarifying which ones were identified [64]. The basic characteristics of the included studies are presented in Table 1. 

### 3.3. Quality Evaluation of the Included Studies

The global quality of the included studies could be characterized as acceptable. All of the included studies, based on their primary research questions, provided clearly stated objectives, and used the appropriate design for their research. Similarly, the definition and representation of the targeted population were both clearly outlined. All of them used established measurements for the variables under investigation. It must be noted that a portion of them used self-report instruments whereas others performed clinical interviews which are judged as superior compered to questionnaires. However, all measurements were suitable for the variables that was intended to be evaluated. The included studies described the methods performed in detail, with a pre-definition of the statistical significance, and provided detailed curation of their data. The discussion sections were all justified given each study’s findings. The quality evaluation revealed a number of drawbacks. There was no reporting on non-respondents and the lack of sample justification emerged as a major issue since none of them performed a sample size calculation. Given the nature of the studies’ design (cross-sectional) this finding could be regarded as alarming, since it creates obstacles in the generalization of the findings [65]. Results of the quality evaluation are presented in Table 2. 

### 3.4. Main Findings Regarding the Research Question 

Regarding affective disorders, results appear to be discrepant. In one of the studies, obesity was significantly correlated with over the cut-off point scores for the diagnosis of depression [56], and this correlation was stronger when participants had higher concerns about their weight. Three more studies of those examining depression and obesity reported higher possibility for an individual to be obese, when depression was present [53,54,55]. With respect to bipolar disorder (BD), individuals with BD had significantly higher possibility of being obese than individuals without BD. Obesity was associated with self-harming behaviors and suicide attempts, and significantly associated with the intake of psychotropic medication, when this did not involve selective serotonin reuptake inhibitors (SSRI’s) [59]. On the contrary, one of the studies regarding depression, found non-significant association between this psychiatric disorder and obesity, except when ethnicity was taken into consideration [51]. The last study, involving a mood disorder, found a significant but weak association between depression and obesity, but only for younger (12–15 years of age) compared to older (15–18 years of age) adolescents [52]. Disorders in the psychotic spectrum and conduct disorder were investigated only in one study each [58,62]. None found significant associations between the disorder and obesity, even though in the case of conduct disorder, when individuals perceived themselves as obese, the symptomatology of the disorder was worse. 

With respect to anxiety disorders, three of the studies examined their relation to obesity without specifying which disorders were examined, and results could be characterized as contradicting. One of the studies found a significantly higher possibility for participants with an anxiety disorder to be obese [61], while the other two found no significant relation [57,60]. However, it must be noted that in the study that did find a correlation, anxiety was evaluated with clinical interviews based on DSM-IV, while in the other two studies evaluation was conducted with the use of self-report instruments, which were less valid [66]. With respect to social anxiety, this was investigated by one study, and results showed that for obese individuals, the higher the BMI the worse the symptomatology of social anxiety. Finally, one study in which psychiatric disorders were not clarified but evaluated with clinical interviews based on the DSM-V criteria found that psychiatric disorders presented more often within the obese group than the overweight or normal-weight group, while a predictive model showed that the presence of a psychiatric diagnosis increased the risk of weight gain by a little more than 26 times [64].

## 4. Discussion

Both obesity and psychiatric disorders are complex states which interweave genetic, biological, psychological, and environmental factors [67]. Psychiatric disorders and obesity are both debilitating conditions that demonstrate increasing prevalence during childhood and adolescence [68,69]. The somatic and psychological changes [70] combined with the constant social transitions [71] that occur during these developmental stages render individuals prone to mental illness and unhealthy lifestyle patterns. The aim of this study was to systematically present all relevant data regarding the associations of psychiatric disorders and obesity in childhood and adolescence. The results of the present review appear as slightly contradicting across different disorders, but with a clear trend showing that the two conditions frequently coexist. 

Regarding affective disorders and obesity, most of the included studies reported a significant association between the two conditions [53,54,55,56,59]. Indeed, the literature has shown that they share a common genetic profile as well as similar behavioral patterns. Both bipolar disorder and depression—either typical or atypical—affect and distort energy, motivation, attitude towards eating, and need for sleep of minors [72]. Thereby, these disorders render patients vulnerable to weight gain. In addition, manifestations of these disorders at a cognitive and behavioral level may complicate and obstruct compliance to health management interventions, such as weight regulation [73]. With respect to bipolar disorder, interestingly obesity was significantly associated with psychotropic medication for bipolar disorder, but only when it did not included SSRI’s (bupropion, mirtazapine, venlafaxine, duloxetine) [59]. This finding contradicts relevant research in an adult sample from a large 10-year cohort study, which included almost 300,000 BMI measurements, that has shown that individuals of normal-weight at baseline were 1.29 times more possible to transit to overweight or obesity, and those with overweight at baseline were 1.29 times more possible to transit to obesity [74]. However, evidence among underaged population is inconsistent. Research has shown that the use of SSRIs in children and adolescents did not cause BMI increase [75,76], while other studies have concluded that antidepressant medication (of any class) for adolescent depression was significantly and independently linked to the weight trajectory [77]. 

Apart from the psychological and behavioral connection, affective disorders also share a common biological profile with weight gain. The most prominent biomarker associated with depression is cortisol, due to the deregulation of the hypothalamic–pituitary–adrenal (HPA) axis [78]. The end product of this deregulation is similar to the outcomes of Cushing syndrome, an endocrinological disorder which is characterized by excessive visceral fat accumulation [66]. Despite the fact that the increase in cortisol level in depression and Cushing’s is different, it has the same impact of central adipose tissue deposition [79]. Another central symptom of depression, sleep disturbance, may increase the possibility of obesity. The two responsible hormones for appetite are leptin and ghrelin [80]. Ghrelin is produced by the gut and triggers consuming behaviors through hypothalamic structures. Leptin, on the other hand, is produced in the periphery and produces the signal of satiety. With a normal circadian rhythm and sleep–wake cycle, leptin is increased and ghrelin is decreased [81]. Thereby, an individual with depression may present with obesity due to disturbed sleep patterns, a state that has been characterized as “leptin resistance” [82]. 

The relation between disorders in the psychotic spectrum and obesity was examined only in one study [58], possibly due to the fact that psychosis emerges during late adolescence and early adulthood [83], and thereby, relevant research is limited. It must be noted that studies have shown that the disorder itself is responsible for glucose deregulation, even though evidence is scarce [84,85,86]. The included study [58] found no difference in BMI between adolescents with psychosis and healthy individuals, contradicting a prospective study that found an increase of more than 7% two years following the first psychotic episode in a sample of individuals 7 to 35 years old [87]. At this point, the role of antipsychotic medication should be discussed. Data on approved psychotropic medication for minors and weight gain risk are presented in Table 3. Though data from populations of minors are limited, research has shown that atypical antipsychotics are responsible for weight gain and linked to metabolic disturbances [88]. For example, one study has found that one-year exposure of children and adolescents to antipsychotic medications was associated with weight gain, and particularly with a mean increase of 11.6 kg [89]. Risperidone is among the most commonly used antipsychotic medications prescribed for children and adolescents [90] with high effectiveness on symptomatology, yet it has been linked to weight gain, hyperglycemia, and diabetes. Notably, one clinical trial comparing risperidone and olanzapine outcomes on pre-school children reported a 2.8 kg weight gain within 6 weeks of medication [91]. Other studies in adolescents receiving antipsychotics conclude that this age group is more vulnerable to weight gain and extrapyramidal symptomatology than adults [92,93]. 

One of the included studies, investigated the connection between social anxiety and obesity, with results indicating that social anxiety and BMI were significantly correlated. Indeed, a relevant study came in similar findings; researchers included 150 adolescents aged between 14 and 18, and found that social anxiety and obesity had a positive relationship [94]. Schachter’s externality theory of obesity supports that obese individuals are less likely to adhere to internal signs of hunger and satiety, but tend to be more susceptible to external food cues when compared to lean counterparts [95]. According to this theory, anxiety and fear may cause an increase in food intake, as eating functions as a soothing mechanism towards intense stress. 

Three of the included studies investigated the correlation between anxiety disorders and obesity, and their results were contradicting. Two found a significant association between increased BMI and the presence of anxiety disorders [61,63], while the other found no significant association [60]. A relevant meta-analysis that explored the association between weight gain and anxiety in adolescence demonstrated a significant relationship, but the magnitude was small [41]. Longitudinal studies have shown that anxiety in underaged samples has been significantly associated with obesity [96]. Again, as in the case of mood disorders, the HPA axis seems to play a central key role in the anxiety–obesity relationship. Elevated glucocorticoids due to anxiety and chronic stress may lead to increased food intake, and more particularly to increased consumption of “comfort food”, which is rich in fat and sugar [97,98]. 

## 5. Conclusions

Understanding the nexus between obesity and psychiatric disorders in children and adolescents is of great importance, given the alarming increase in both conditions in underaged populations [98]. This will facilitate targeting the most critical factors when developing and implementing relevant interventions. The main contribution of the present study lies in the fact that it sets the behavioral ground for both mental health professionals and weight management experts to intervene when assessing obese minors with psychiatric comorbidities. In addition, it highlights the role of pharmaceutical interventions, which should be taken into consideration when BMI is alarming. 

This systematic review bears certain limitations that need to be addressed. Firstly, the included studies were of cross-sectional design. It has been well documented that observational research, and particularly of cross-sectional design, is prone to specific biases, such as the lack of evaluation of mediating factors to which the results could be attributed and are not assessed [99]. Moreover, many of the included studies evaluated the psychiatric disorders with self-report instruments rather than clinical assessments. The credibility of this method has been questioned for psychiatric diagnoses [100]. The limited research regarding different psychiatric diagnoses could be perceived as an additional limitation of this study. Most of the studies focused on depression and anxiety, rather than more severe diagnoses, such as psychosis. However, this could be attributed to the fact that samples included adolescents and children; many severe mental illnesses have their onset during late adolescence and early adulthood. The proportion of patients diagnosed with a mental disorder before the age of 18 is almost 40% and the most common disorders diagnosed before that age are neurodevelopmental (83.2%), anxiety/fear-related (51%), eating disorders (48.1%), and obsessive–compulsive/related disorders (45.1%) [101]. Lastly, most of the included studies did not report on whether participants were medication-naïve or not, and thereby, this fact could have affected the results. 

## Figures and Tables

**Table 1:** Basic characteristics of the included studies.

First Author [Reference]	Publication Year	Sample Characteristics [Number of Participants (Mean Age ± SD or Age Range in Years) % Females]	Primary Diagnosis	Diagnosis Establishement	Main Results
Assari and Caldwell [51]	2015	1170 (13–17) 52%	Depression	CIDI	Non-significant associations between obesity and MDD, significant interaction between ethnicity and obesity in MDD.
Chen [52]	2015	1101 (12–18) 45.8%	Depression	CES-D	Depression was positively but weakly associated with obesity in younger adolescents (12–15 years), but not in older adolescents (15–18 years).
Hoare [53]	2014	800 (13.1 ± 0.62) 55%	Depression	SMFQ	Obesity contributed significantly to depressive symptomatology, and some of these patterns differed across gender. Adolescents with greater sedentary behaviors reported greater symptomatic depression, before and after adjusting for covariates.
Lindberg [54]	2020	12507 (6–18) 46.9%	Depression, anxiety	ICD-10	Obesity was a significant risk factor for anxiety and depression in children and adolescents. Obese girls had 43% higher risk for anxiety and depression (*p* < 0.0001). The risk in obese boys was similar (*p* < 0.0001).
Rottenberg [55]	2014	566 (7–14) NA	Depression	SCA-D	Individuals with a diagnosis of depression had higher rate of obesity than controls (OR 3.67, CI = 1.42–9.52).
Ting [56]	2012	869 (15.7) 47%	Depression	CES-D	Depressive symptomatology significantly correlated with obesity (*p* = 0.02) and the association was mediated by perceived weight status, increased weight concern, and food uptake restraint.
Moharei [57]	2018	160 (5–17) 47.5%	Anxiety, depression	STAIC, CDI	Non-significant differences in scores of anxiety and depression between obese and non-obese individuals.
Jensen [58]	2017	113 (15.74 ± 1.36) 69.9%	Schizophrenia	ICD-10	BMI did not differ between patients on first psychotic episode without antipsychotic treatment and controls (matched for sex, age, and parental education level).
Shapiro [59]	2016	118 (16.0 ± 1.5) 62%	Bipolar disorder	KSADS-PL	Adolescents with bipolar disorder reported significantly higher obesity (18%) than controls (*p* = 0.02). Among those with psychiatric diagnosis, obesity was significantly associated with suicide attempt and self-injurious behavioral patterns. Antidepressants were associated with obesity, but only when medication did not involve selective serotonin reuptake inhibitors.
Lim [60]	2015	199 (7–12) NA	Anxiety	CBCL	Children in the Obesity + Clinical Anxiety group reported more body dissatisfaction (*p* = 0.023) and lower total HRQOL (*p* = 0.005) than the control group.
Roberts [61]	2016	3134 (11–17) 48.8%	Anxiety	DSM-IV	Significant association of anxiety with obesity. Any anxiety disorder was associated with a 46% increased possibility to be obese.
Lankinen [62]	2017	2275 (15.6 ± 0.4) 48.9%	Depression, conduct disorder, social anxiety	BDI, SPIN, YSR	Perceived weight status was correlated with higher risk of self-reported depression in girls (*p* < 0.001) and boys (*p* = 0.001). Significant association was also found for social phobia (*p* = 0.05) in boys.
Thompson [63]	2012	230 (<17) 44.5%	Social anxiety	SAS	Social anxiety was significantly and positively correlated with BMI. Extremely obese participants scored significantly higher in the social anxiety scale than obese.
Rojo [64]	2021	200 (10.34 ± 1.31) 60%	Psychological stress events, psychiatric diagnoses	DSM-5 clinical interviews	Obese children presented a psychiatric disorder more often than overweight or normal-weight children. A predictive model revealed that a psychiatric diagnosis increased the risk of weight gain by 26.

ABBREVIATIONS: NA = Not applicable; SD = Standard Deviation; CIDI = World Mental Health Composite International Diagnostic Interview; CES-D = Center for Epidemiologic Studies-Depression Scale; SMFQ = Short Moods and Feelings Questionnaire; ICD-10 = International Classification of Diseases, 10th revision; SCA-D = Schedule for Children and Adolescents-Diagnostic Version; KSADS-PL = Kiddie Schedule for Affective Disorders and Schizophrenia for School Age Children, Present and Lifetime version; BDI = Beck Depression Inventory; BMI = Body Mass Index; SPIN = Social Phobia Inventory; YSR = Youth Self Report; SDQ = Strengths and Difficulties Questionnaire; LSAS = Liebowitz Social Anxiety Scale; CBCL = Anxiety Problems scale from the Child Behavior Checklist; STAIC = State-Trait Anxiety Inventory; CDI = Children’s Depression Inventory.

**Table 2:** Quality assessment of included studies using the AXIS tool.

	Study Reference													
AXIS Item	51	52	53	54	55	56	57	58	59	60	61	62	63	64
Clearly stated objective	Y	Y	Y	Y	Y	Y	Y	Y	Y	Y	Y	Y	Y	Y
Appropriate study design	Y	Y	Y	Y	Y	Y	Y	Y	Y	Y	Y	Y	Y	Y
Justified sample size	N	N	N	N	N	N	N	N	N	N	N	N	N	N
Clearly defined population	Y	Y	Y	Y	Y	Y	Y	Y	Y	Y	Y	Y	Y	Y
Clearly represented population	Y	Y	Y	Y	Y	Y	Y	Y	Y	Y	Y	Y	Y	Y
Clear selection process of population	Y	Y	Y	Y	Y	Y	Y	Y	Y	Y	Y	Y	Y	Y
Address and categorize non-responders	N	N	N	N	N	N	N	N	N	N	N	N	N	N
Appropriate variable measurement	Y	Y	Y	Y	Y	Y	Y	Y	Y	Y	Y	Y	Y	Y
Use of established measurements	Y	Y	Y	Y	Y	Y	Y	Y	Y	Y	Y	Y	Y	Y
Reported statisticalsignificance	Y	Y	Y	Y	Y	Y	Y	Y	Y	Y	Y	Y	Y	Y
Methods sufficiently described	Y	Y	Y	Y	Y	Y	Y	Y	Y	Y	Y	Y	Y	Y
Data description	Y	Y	Y	Y	Y	Y	Y	Y	Y	Y	Y	Y	Y	Y
Concerns about non-response bias?	Y	Y	Y	Y	Y	Y	Y	Y	Y	Y	Y	Y	Y	Y
Information about non-responders	N	N	N	N	N	N	N	N	N	N	N	N	N	N
Internal consistency of results	Y	Y	Y	Y	Y	Y	Y	Y	Y	Y	Y	Y	Y	Y
Adequate result presentation	Y	Y	Y	Y	Y	Y	Y	Y	Y	Y	Y	Y	Y	Y
Justified results by discussion	Y	Y	Y	Y	Y	Y	Y	Y	Y	Y	Y	Y	Y	Y
Report on limitations	Y	Y	Y	Y	Y	Y	Y	Y	Y	Y	Y	Y	Y	Y

Y = yes; N = no; DK = do not know.

**Table 3:** Approved psychiatric medication for minors and weight gain risk.

Pediatric Labeling Aproval Date	Pharmaceutical Substance	Indication	Therapeutic Category	Weight Gain Risk
28/1/2022	ziprasidone	BD-I (10 to 17 years)	SGA	Low
27/12/2021	brexpiprazole	Schizophrenia (13 to 17 years)	SGA	Low
5/3/2018	lurasidone	Treatment of MDE associated with BD-I	SGA	Low
27/1/2017	lurasidone	Treatment of schizophrenia in adolescents and irritability associated with autistic disorder in pediatric patients	SGA	Low
12/3/2015	asenapine	Schizophrenia and Acute Manic or Mixed Episodes Associated with BD-I	SGA	Moderate
31/10/2014	escitalopram	MDD	SSRI	Moderate
16/10/2014	duloxetine	GAD	SNRI	Low
26/7/2013	olanzapine/fluoxetine	Depressive episodes associated with BD-I	SGA/ SSRI	High
30/4/2013	quetiapine	Bipolar depression	SGA	Moderate
18/10/2012	duloxetine	MDD	SNRI	Moderate
2/12/2009	quetiapine	Schizophrenia (13 to 17 years) and bipolar mania (10 to 17 years)	SGA	Moderate
19/3/2009	escitalopram	MDD in adolescents	SSRI	Moderate
14/8/2008	olanzapine	schizophrenia; BD	SGA	High
27/2/2008	aripiprazole	BD-I	SGA	Moderate
29/10/2007	aripiprazole	Schizophrenia	SGA	Moderate
22/8/2007	risperidone	Schizophrenia;short-term treatment of acute manic or mixed Episodes associated with BD-I	SGA	High
18/2/2005	citalopram	MDD	SSRI	Moderate
18/2/2005	sertraline	MDD & OCD	SSRI	Low
12/1/2005	paroxetine	MDD	SSRI	Low
12/1/2005	mirtazapine	MDD	NaSSA	High
12/1/2005	nefazodone	MDD	SARI	Low
5/5/2004	venlafaxine	MDD	SNRI	Low
3/1/2003	fluoxetine	MDD & OCD	SSRI	Low
19/7/2001	buspirone	GAD	Anti-Anxiety Agents/Anxiolytics	Low

ABBREVIATIONS: BD-I = Bipolar Disorder I; MDE = Major Depressive Episode; MDD = Major Depressive Disorder; OCD = Obsessive Compulsive Disorder; GAD = Generalized Anxiety Disorder; SGA = Second-generation antipsychotic; SSRI = Selective serotonin reuptake inhibitor; SNRI = Serotonin and norepinephrine reuptake inhibitor; NaSSA = Noradrenergic and specific serotonergic antidepressant. NOTES: Data on medication have been drawn from the FDA (https://www.fda.gov/science-research/pediatrics/pediatric-labeling-changes). Weight gain risk was assessed based on studies on underaged populations. When these were not available, assessment was based upon adult populations.

## Data Availability

Not applicable.

## Supplementary Materials

The following supporting information can be downloaded at: https://www.mdpi.com/article/10.3390/children10020285/s1. PRISMA Checklist. Reference [102] is cited in the Supplementary Materials.
